# High Enzyme Activity of a Binuclear Nickel Complex Formed with the Binding Loops of the NiSOD Enzyme**

**DOI:** 10.1002/chem.202002706

**Published:** 2020-11-09

**Authors:** Dóra Kelemen, Nóra V. May, Melinda Andrási, Attila Gáspár, István Fábián, Norbert Lihi

**Affiliations:** ^1^ Department of Inorganic and Analytical Chemistry University of Debrecen 4032 Debrecen Hungary; ^2^ Research Centre for Natural Sciences 1117 Budapest Hungary; ^3^ MTA-DE Redox and Homogeneous Catalytic Reaction Mechanisms, Research Group University of Debrecen 4032 Debrecen Hungary

**Keywords:** coordination modes, EPR spectroscopy, metallopeptides, nickel, redox chemistry, superoxide anions

## Abstract

Detailed equilibrium, spectroscopic and superoxide dismutase (SOD) activity studies are reported on a nickel complex formed with a new metallopeptide bearing two nickel binding loops of NiSOD. The metallopeptide exhibits unique nickel binding ability and the binuclear complex is a major species with 2×(NH_2_,N_amide_,S^−^,S^−^) donor set even in an equimolar solution of the metal ion and the ligand. Nickel(III) species were generated by oxidizing the Ni^II^ complexes with KO_2_ and the coordination modes were identified by EPR spectroscopy. The binuclear complex formed with the binding motifs exhibits superior SOD activity, in this respect it is an excellent model of the native NiSOD enzyme. A detailed kinetic model is postulated that incorporates spontaneous decomposition of the superoxide ion, the dismutation cycle and fast redox degradation of the binuclear complex. The latter process leads to the elimination of the SOD activity. A unique feature of this system is that the Ni^III^ form of the catalyst rapidly accumulates in the dismutation cycle and simultaneously the Ni^II^ form becomes a minor species.

## Graphical abstract

**Nickel binding complexes**: A nickel complex formed with a new metallopeptide bearing twin nickel binding loops of NiSOD exhibits unique nickel binding ability. The binuclear complex is a major species even in an equimolar solution. The complex exhibits superior superoxide dismutase (SOD) activity. A unique feature of this system is that the Ni^III^ form of the catalyst rapidly accumulates in the dismutation cycle and the Ni^II^ form simultaneously becomes a minor species.

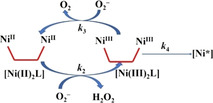

## Introduction

The superoxide anion radical (O_2_^−^) is an unavoidable, highly reactive intermediate in the biochemistry of aerobic organisms. It is present at low concentration levels regulated by superoxide dismutase enzymes (SODs).[^1^, ^2^] In the absence of such enzymes, the elevated level of reactive oxygen species (ROS) causes significant oxidative stress and cellular damage.^[3]^ The decomposition reaction of the superoxide anion radical yields O_2_ and H_2_O_2_; the latter is degraded to harmless products through various pathways.[^4^, ^5^] The SODs are classified into four classes in accordance with the metal ion in their active center. These are the copper/zinc, iron, manganese, and the recently discovered nickel containing SOD enzymes (NiSOD). The latter enzyme is expressed by the *sodN* gene and found in several marine cyanobacteria and *Streptomyces* species.^[6]^ The NiSOD enzyme is built in homohexameric form consisting four helix‐bundle subunits.^[7]^ Each monomer contains the catalytically active nickel ion, and the dismutation reaction occurs through a proton‐coupled electron‐transfer mechanism accompanied with the cycling of the oxidation state of nickel between +2 and +3.[^8^, ^9^] Nickel is coordinated in the N‐terminal part of the peptide. This coordination motif features a loop within the first six amino acid residues (HCDLPC). Earlier studies have demonstrated that this sequence is the minimal peptide motif for mimicking the catalytic cycle as well as the binding mode of NiSOD.^[10]^ In the case of reduced form, nickel(II) is accommodated by the binding of the terminal amino group, the peptide nitrogen and the two thiolates of cysteine residues. It is a square‐planar diamagnetic complex. The oxidation leads to the rearrangement of the coordination sphere, and the axial position of the paramagnetic nickel(III) is bound via the imidazole‐*N* of histidine in a square‐pyramidal coordination environment.^[11]^ In our previous work, detailed equilibrium and spectroscopic results were reported on nickel(II) complexes formed with some NiSOD model peptides.^[12]^ The results have clearly shown that the acetylation of the terminal group significantly reduces the nickel binding affinity,^[13]^ and the oxidation yields nickel(III) coordination isomers. The role of the cysteine fragments was also investigated and spectroscopic studies unambiguously confirmed that the presence of cysteine in the secondary position is crucial to establish the square‐planar geometry, while the distant cysteine may alter the redox properties of the Ni^II^/Ni^III^ couple.^[14]^ Nevertheless, both cysteine residues are essential for the efficient dismutation of superoxide ion.^[15]^

**Scheme 1:**
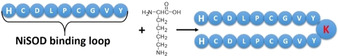
Anchoring two binding motifs of the NiSOD enzyme by lysine.

The coordination ability and catalytic features of multiple NiSOD binding loops have not been investigated before. In this work, we report detailed equilibrium, spectroscopic, and SOD activity studies on the new metallopeptide formed with a peptide (denoted as **L** thorough this paper) containing twin binding loops of the NiSOD enzyme. In this ligand, a lysine scaffold provides two amino groups to anchor the two HisCysAspLeuProCysGlyValTyr binding motifs (Scheme 1 and Scheme S1).

## Results and Discussion

### Acid‐base equilibria

The acid dissociation constants of the ligand were determined by pH‐potentiometry and are collected in Table 1. Some of the stepwise acid‐base processes overlap significantly. The two well‐defined equivalence points in the titration curve offers a possibility to determine the exact concentration of the stock solution of the ligand. The same titration curve is also suitable for the calculation of the acid dissociation constants of the ligand (Figure S1). In accordance with the structure of the ligand, the terminal amino groups, the carboxylate groups of the *C*‐termini and the aspartic acids, the thiols of cysteines, the hydroxyl groups of tyrosine residues as well as the two imidazole rings of histidine moieties take part in the acid‐base processes. The fitting of the titration curve confirms that 13 acid dissociation constants can be estimated, which is consistent with the expected number of acid‐base processes. Detailed analysis of the data indicates that the lowest log *K*_i_ values are associated with the carboxylic groups of the *C*‐termini and the side chains of aspartic acid residues. The further deprotonation steps occur on the imidazole rings with increased acidity at the *N*‐terminus. This effect can easily be explained by the intramolecular hydrogen bond between the terminal amino group and imidazole‐*N* of histidine, which increases the acidity of imidazoles and separate it from the subsequent deprotonation processes. Upon increasing the pH, the further deprotonation steps overlap significantly. On the basis of reported data, it is reasonable to assume that the lower log *K*_i_ values belong mainly to the ammonium functions of the *N*‐terminus and to the thiolates of the cysteine residues,^[16]^ while the two highest acid dissociation constants are assigned to the deprotonation of hydroxyl groups of tyrosine moieties.^[12]^

**Table 1:** The protonation constants (log β_qr_) of **L** and the stability constants (log *β*_pqr_) of the complexes formed between Ni^II^ and **L**.^[a,b]^

Species	log *β*_qr_	Species	log *β*_qr_
H**L**	10.03(2)	H_12_ **L**	88.3(1)
H_2_ **L**	19.89(4)	H_13_ **L**	91.6(1)
H_3_ **L**	29.12(3)	NiH_6_ **L**	64.6(1)
H_4_ **L**	37.86(7)	NiH_4_ **L**	50.5(1)
H_5_ **L**	46.29(4)	Ni**L**	13.8(1)
H_6_ **L**	54.25(7)	NiH_−1_ **L**	4.7(1)
H_7_ **L**	61.61(5)	Ni_2_H_6_ **L**	67.5(6)
H_8_ **L**	68.86(6)	Ni_2_H_4_ **L**	56.8(3)
H_9_ **L**	74.86(7)	Ni_2_ **L**	29.6(3)
H_10_ **L**	80.28(8)	Ni_2_H_−2_ **L**	8.9(1)
H_11_ **L**	84.69(1)		

log $K_{NiH_{6}L}^{{Ni}_{2}H_{6}L}$	2.9		
log $K_{NiH_{4}L}^{{Ni}_{2}H_{4}L}$	6.3		
log $K_{NiL}^{{Ni}_{2}L\;}$ ^[c]^	−2.0		

[a] *I*=0.2 m KCl, *T=*298 K. [b] 3σ standard deviations are indicated in parentheses. [c] *K*_NiL_=*β*_NiL;_ *K*_Ni2L_=*β*_Ni2L_/*β*_NiL_=[Ni_2_ **L**]/([Ni**L**][Ni]).

### Nickel(II) complexes

**Figure 1:**
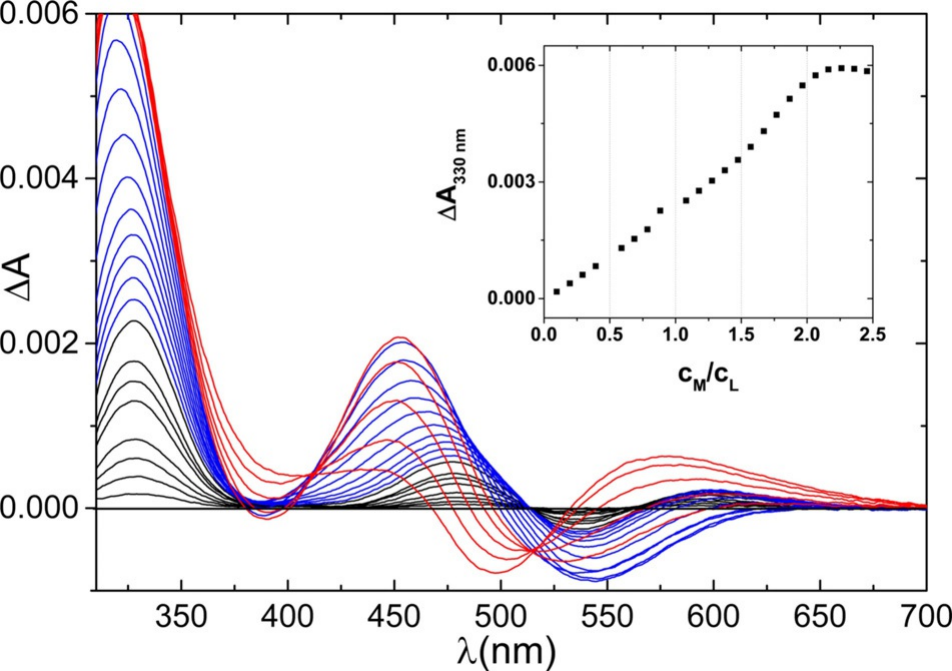
CD titration of **L** with Ni^II^ at pH 7.6. The spectra in black correspond to the samples between 0 and 1 equivalent of Ni^II^ per **L**, the spectra in blue correspond to the samples between 1 and 2 equivalent of Ni^II^ per **L** and the spectra in red correspond to the samples between 2 and 2.5 equivalent of Ni^II^ per **L**. Inset: evolution of Δ*A* at 330 nm. *c*_**L**_=1.1 mm.

The metal ion binding properties of **L** were studied by CD spectroscopic titration. Aliquots of NiCl_2_ were added to the solution of **L** in HEPES buffer (10 mm, pH 7.6, *I*=0.2 m (KCl)) between 0 and 2.5 equivalent per **L** and the spectra were recorded (Figure 1). As expected, the complex formation with nickel(II) leads to significant CD activity both in the UV and visible regions of the spectra. The addition of nickel(II) results in the appearance of three bands up to 1:1 Ni^2+^ to ligand ratio. The spectrum features a dominant transition at 330 nm (+), as well as two other transitions at 478 nm (+) and 544 nm (−), which are assigned to the S^−^→Ni^II^ LMCT and the *d–d* transitions, respectively. These results confirm that the complex has square‐planar geometry. The addition of further Ni^II^ leads to a slight shift in the visible segment of the spectra and to an increase in the intensity of the UV band, indicating the formation of a new complex. A plateau is observed in the intensity of the S^−^→Ni^II^ LMCT band after adding more than 2 equivalents of Ni^II^ and the pronounced change in the *d*–*d* part of the spectra indicates the reorganization of the coordination sphere. This effect clearly shows the binding of the third nickel(II) ion. In accordance with plausible considerations, the coordination of further amide nitrogen atoms of the peptide backbone are expected. Moreover, the spectral changes correlate well with the formation of Ni**L** and Ni_2_ **L** species, which corroborates the postulated equilibrium model discussed in the subsequent part of the paper (Figure S2).

**Figure 2:**
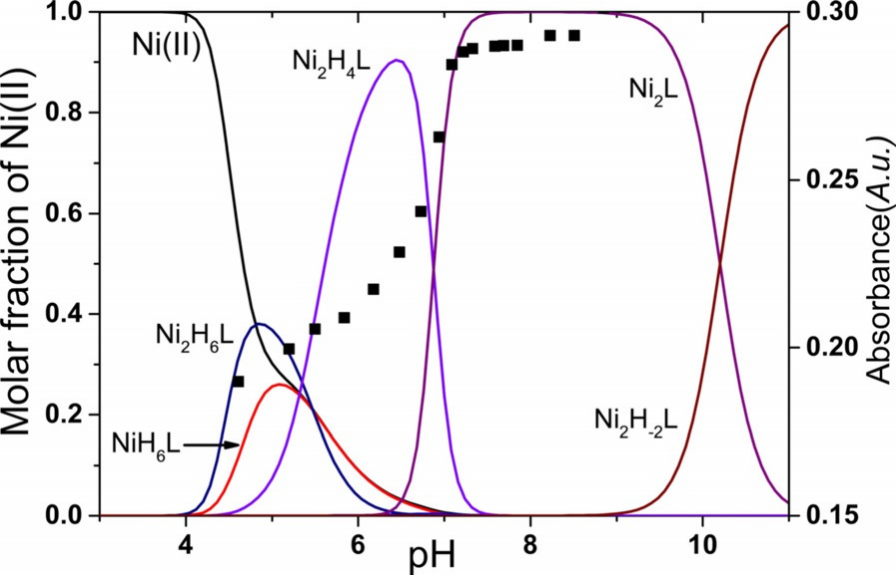
Distribution of the complexes formed in the Ni^II^/**L** 2:1 system and the absorbance at 450 nm obtained by UV/Vis spectroscopy as a function of pH (*I*=0.2 m KCl, *T=*298 K). *c*_Ni(II)_=2.4 mm.

The complex formation processes between nickel(II) and the ligand were studied at 1:1 and 2:1 metal to ligand ratios (the corresponding titration curve obtained at 2:1 metal to ligand ratio is available in the Supporting Information, Figure S3). The stability constants of the corresponding complexes were determined by pH‐potentiometric titrations and the data are collected in Table 1. Since the complex formation processes between nickel(II) and **L** are relatively slow (see below), care was taken to ensure that the equilibrium was established for each point during the pH‐potentiometric titrations (see details in the Supporting Information). The concentration distribution at 2:1 metal to ligand ratio and the absorbance at 450 nm are shown as a function of pH in Figure 2. The spectral effect corresponds to the *d*–*d* transition of square‐planar nickel(II) complexes.

The complexation starts under slightly acidic conditions with the formation of histamine‐like coordinated species (Ni_2_H_6_ **L**) and an additional base consumption process yields the binding of the thiolate groups (Ni_2_H_4_ **L**). The formation of a macrochelate coordinated complex is expected and nickel(II) is bound via the 2×(2N,2S) donor set. This species exhibits significant CD activity, which is characteristic for square‐planar geometry (Figure S4).

Upon increasing the pH, the next process leads to the formation of the active site of NiSOD with the two (NH_2_,N_amide_,S^−^,S^−^) donor sets (Figure S5, Ni_2_ **L**). Both the UV/Vis and CD spectroscopic data are in good agreement with those observed for the wild‐type fragment of NiSOD (Figure S4, S6).^[12]^ Unexpectedly, highly stable binuclear complexes form with this coordination motif. This effect manifests itself in the ratio of the stepwise stability constants, which is considerably smaller than 1, log (*K*_Ni**L**_/*K*_Ni2**L**_)≈−2.0 (Table 1). This is a clear indication that the formation of the binuclear complex is highly preferred even in an equimolar solution of the ligand and the metal ion. Moreover, it confirms the cooperativity between the two nickel(II) centers; that is, the binding of the first nickel(II) ion induces further structural changes, which is favorable to accommodate the second metal ion. Such cooperativity is not observed in the case of the corresponding protonated complexes for example, Ni_2_H_6_ **L**/NiH_6_ **L**. These complexes are formed under acidic conditions and the protonation of several donor groups likely makes the necessary rearrangement of the ligand unfeasible.

To confirm this unique equilibrium feature, CE‐MS experiments were carried out at 0.9:1 metal to ligand ratio at pH 9.0. As expected, the extracted ion electropherogram shows three peaks (Figure S7). The MS spectra associated with these peaks confirms the existence of **L**, Ni**L** and Ni_2_ **L** (Figure S8, S9 and S10). Detailed analysis of the isotope patterns also reveals the presence of another species in each case, which is characterized with a very similar set of MS peaks to the noted compounds but at two units smaller *m*/*z* values. This specific feature is likely due to the ESI induced dehydrogenation of the tyrosine moiety of the free‐ as well as the coordinated ligand.[^12^, ^17^] The excellent match of the experimental and calculated isotope patterns corroborates this conclusion. Above pH 9.5, the deprotonation of noncoordinating tyrosine residues leads to the formation of Ni_2_H_−2_ **L**_._

**Figure 3:**
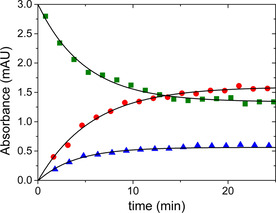
The decay of the concentration of the free ligand (▪) and simultaneous formation of complexes Ni**L** (•) and Ni_2_ **L** (▴) as a function of time at 0.9:1 metal to ligand ratio (pH 9.0). These time profiles were recorded in designated CE experiments (cf. experimental details) and were fitted by a single exponential function (solid line). *c*_L_=42 μm.

The unique nickel binding ability of the ligand can readily be explained by considering that the binding of the first nickel ion stabilizes the structure of the ligand for a favorable coordination of the second nickel ion. In a set of CE experiments, the metal ion and the ligand were mixed, and the samples were injected into the CE instrument by systematically increasing the incubation time of the reaction mixture. The first point could be recorded about 1 min after mixing. The intensities of the separated peaks are plotted as a function of time in Figure 3. Each kinetic curve can be fitted to a simple exponential expression and the corresponding first‐order rate constants (0.20–0.25 min^−1^) are in reasonable agreement. Given the limitations of the experimental arrangement, these results need to be considered semi‐quantitative. Nevertheless, they provide essential information on the mechanism of the complex formation. It is reasonable to assume that the structural rearrangement of the ligand is the rate‐determining step. The simple first‐order characteristics exclude the possibility that the metal ion assists this process in any way. Instead, different forms of the ligand may exist in equilibrium and Ni^II^ coordinates quickly to one of these isomers. The coordination of the first metal ion shifts the equilibrium between the isomers (a relatively slow first‐order process) and also induces further structural changes for binding quickly the second metal ion. Since Ni**L** and Ni_2_ **L** form at about the same rate, the equilibrium between these two species needs to be established relatively fast, but not fast enough to average the corresponding peaks in the CE experiments.

### Nickel(III) complexes of the model peptide

**Figure 4:**
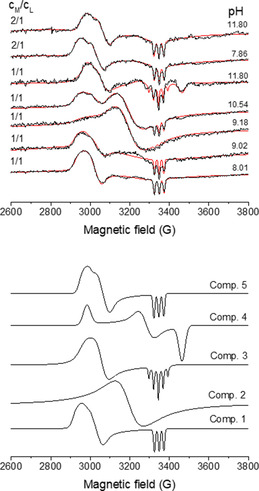
Top: X‐band EPR spectra recorded in the Ni^III^/**L** system after in situ oxidation (black) recorded at 77 K and the simulated EPR spectra (red) after superposition of the individual spectra. Bottom: Individual spectra of the nickel(III) complexes.

To model the enzyme mimetic behavior, the feasibility of the oxidation of the nickel(II) complexes was investigated. KO_2_ was used as an oxidizing agent and the in situ generated Ni^III^ complexes were characterized by EPR spectroscopy. The *g* values and the hyperfine tensors are collected in Table S1. In general, the structures of the Ni^III^ transient species strongly depend on the metal to ligand ratio. At 2:1 metal to ligand ratio, both nickel(II) are accommodated by the (NH_2_,N_amide_,S^−^,S^−^) donor set and the oxidation yields an exclusive Ni^III^ species (Figure 4 Component 1). In this complex, the axial position of Ni^III^ is bound via the imidazole‐*N* of histidine (Figure S11). This binding mode leads to a hyperfine interaction between the unpaired electron on the *d* ${}_{z{}^{2}}$ orbital and the imidazole nitrogen. Since the coordination motifs of the two metal binding sites are exactly the same, the formation of a binuclear species with identical nickel(III) centers is expected under the conditions of EPR spectroscopy.

The corresponding EPR parameters are similar to those observed for NiSOD (*g_x_*=2.289, *g_y_*=2.220, *g*_z_=2.012; *a*_*x,y*_^N^=17.5 G, *a*_z_^N^=25.2 G).[^7^, ^12^] On the basis of these parameters, it is reasonable to assume that the two NiSOD binding motifs act as an independent metal‐binding site and each metal center is capable of promoting the degradation of superoxide ion. In addition, weak dipolar interactions are plausible between the two Ni^III^ centers. This effect is shown in the orientation‐dependent linewidth parameters (Table S1). These parameters were calculated for the wild‐type fragment of NiSOD and **L** and the concomitant increase in *δ_x_* and *δ_y_* confirms such dipolar interactions. At 1:1 metal to ligand ratio, the observed EPR spectra cannot be assigned to one rhombic *g*‐tensor, consequently, the oxidation leads to the formation of coordination isomers. At physiological pH, the oxidation yields the same Ni^III^ transient species that is observed at 2:1 metal to ligand ratio (Component 1). Upon increasing the pH, rearrangement of the coordination sphere was observed during the oxidation. This effect is clearly seen at pH 9.18, where the broad signal provides unequivocal evidence for the dipole–dipole interactions between the paramagnetic Ni^III^ centers (Component 2). The rearrangement is complete in strongly alkaline solution and the EPR parameters can be fitted well by assuming the presence of two components in 2:1 isomer ratio (Component 3 and 4). For the major species, the *g*_x_ and *g*_y_ parameters are similar to those observed for the wild‐type fragment of NiSOD; however, the superhyperfine splitting clearly shows that two imidazole nitrogen atoms are bound to the axial positions (Component 3). For the minor species, the EPR parameters confirm that the unpaired electron is located in the *d_xy_* or d${}_{x{}^{2}-y{}^{2}}$ orbital and the coordination of the donor groups provides strong π back‐donation (Component 4). Such coordination mode is envisioned when the thiolate groups as well as the amide nitrogen coordinate to Ni^III^ in an elongated octahedral crystal field.[^18^, ^19^]

### Catalytic activity

The SOD activity of the Ni_2_ **L** complex was studied by monitoring the catalytic decay of the superoxide ion at 260 nm. The inherent experimental difficulty with these studies is that the preparation of stable aqueous O_2_^−^ stock solution is not feasible. To circumvent this problem, KO_2_ was dissolved in dimethyl sulfoxide (DMSO) and the kinetic experiments were carried out in 1:1 DMSO/water mixture. Preliminary observations confirmed that mixing water with DMSO is not instantaneous in a simple stopped flow experiment; that is, the spectral disturbances fade away several ten milliseconds after mixing. This artifact was avoided by using the sequential stopped‐flow method. First, a DMSO solution of KO_2_ was mixed with water in 1:1 ratio. After 40 s incubation time, this mixture was mixed with the Ni_2_ **L** complex dissolved in buffered 1:1 DMSO/water solvent.

The spontaneous decomposition of the superoxide ion was studied by using the same experimental protocol in the absence of the catalyst. In this case, a simple second‐order decay was observed. The corresponding second‐order rate constant was obtained by fitting the kinetic traces to the corresponding kinetic expression. The result, *k*_1_=(3.86±0.06)×10^4^ m ^−1^ s^−1^, is in excellent agreement with those reported in the literature.[^20^, ^21^]

**Figure 5:**
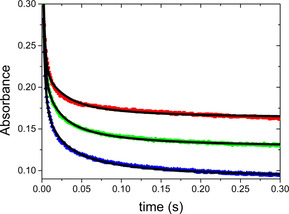
The decomposition of superoxide anion in the presence of Ni_2_ **L** complexes (red: 10 μm; green: 7.55 μm; blue: 5 μm). The kinetic traces were recorded by using the sequential stopped‐flow method. Solid lines represent the fitted kinetic traces on the basis of the proposed kinetic model. Solvent 1:1 aqueous buffer of HEPES (20 mm, pH 7.8)/DMSO mixture. *λ*=260 nm, 2 mm optical path, *c*O_2_^−^=877 μm.

**Scheme 2:**
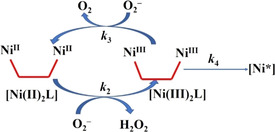
The dismutation reaction of superoxide ion and the degradation of the binuclear nickel complex.

In the presence of the Ni_2_ **L** complex, a very fast decay of the absorbance was observed in the first 30 ms (Figure 5). The absorbance drops from its initial value (ca. 0.5) to below 0.3 in the first measured point (at 2 ms) of these kinetic traces. This clearly demonstrates that a substantial part of the reaction proceeds within the dead‐time of the instrument (ca. 1.5 ms). Since the spontaneous decay of O_2_^−^ occurs on a considerably longer time‐scale, these results suggest that the Ni_2_ **L** complex features high SOD activity. If the dismutation reaction occurred only, a relatively simple first‐order expression could be derived for the interpretation of the data assuming that steady‐state conditions apply for the Ni^III^ complex. Furthermore, the dominant absorbing species should be the original Ni^II^ complex once the dismutation is complete and the absorbance should not change. In reality, the kinetic traces cannot be fitted to a simple first‐order expression, the absorbance is considerably higher than expected at 300 ms and it steadily changes in a slow process. The observations are consistent with a reaction scheme that incorporates the dismutation steps and the kinetically coupled degradation of the nickel complex (Scheme 2). It is not clear whether one or both nickel ions are involved in these reactions. Scheme 2 postulates the formation of Ni^III^_2_ **L** but the same considerations hold if a Ni^II^(Ni^III^)**L** complex is active in the process. Since the two metal centers are essentially identical, their kinetic and redox properties are expected to be very similar. Thus, the dismutation and the degradation may simultaneously occur at both sites at about the same rates and the rate constants in the model stand for the sum of the individual rate constants of the analogous reactions occurring at each metal center.

Experimental evidence is not available to make any distinction between these possibilities and to separate the corresponding processes. In the absence of an oxidant, the Ni^II^_2_ **L** complex is stable, thus, it is reasonable to assume that the degradation involves the oxidized Ni^III^_2_ **L** form. In this species, Ni^III^ may oxidize one of the thiol groups in a fast intramolecular redox step, which is presumably first order for Ni^III^_2_ **L**. The reaction is irreversible and yields an unidentified oxidized complex (Ni*).

In an attempt to identify Ni*, the Ni_2_ **L** complex was treated with KO_2_ and the mass spectrum of the reaction mixture was immediately recorded. According to these MS experiments, the oxidation yields the corresponding sulfoxide, sulfone and sulfonic acid derivatives. The isotope patterns confirmed that the two nickel ions are still bound to the peptide in the oxidized products. Similar products have already been reported in the oxidation of some NiSOD related peptides.^[9]^ Another part of the mass spectrum shows that the oxidation results in the loss of the hydrogen sulfide moieties. Such peptide decay was also observed in our previous work.^[12]^ It is remarkable that the S−S bond formation was not observed in the oxidation process. According to MS experiments, both S‐oxidation and hydrogen sulfide loss are plausible. Such kinetic features have already been proposed for several NiSOD mimetics in the literature; however, detailed analysis of the experimental kinetic data has not been reported before.^[9]^ In accordance with Scheme 2, the detailed kinetic model includes reactions 1, 2, 3, 4 and the corresponding differential equation system is given by Equations 5, 6, 7, 8, 9.

(1) $${2\;O}_{2}^{-}+{2\;H}^{+}=H_{2}O_{2}+O_{2}\;\;\;k{}_{1}$$

(2) $${Ni\left(II\right)}_{2}L+{2\;O}_{2}^{-}+{4\;H}^{+}={Ni\left(III\right)}_{2}L+{2\;H}_{2}O_{2}\;\;\;k{}_{2}$$

(3) $${Ni\left(III\right)}_{2}L+{2\;O}_{2}^{-}={Ni\left(II\right)}_{2}L+{2\;O}_{2}\;\;\;k{}_{3}$$

(4) $${Ni\left(III\right)}_{2}L\to Ni{}^{*}\;\;\;k{}_{4}$$

(5) $$\frac{d\left[O_{2}^{-}\right]}{dt}=\;-\;k_{1}\times [O_{2}^{-}{]}^{2}\;{-\;k}_{2}\times \left[Ni{\left(II\right)}_{2}L\right]\left[O_{2}^{-}\right]-k_{3}\times [Ni(III{)}_{2}L]\left[O_{2}^{-}\right]$$

(6) $$\frac{d\left[H_{2}O_{2}\right]}{dt}=\;k_{2}\times \left[Ni{\left(II\right)}_{2}L\right]\left[O_{2}^{-}\right]\frac{d\left[Ni\left(II\right)\right]}{dt}=\;-k_{2}\times \left[Ni{\left(II\right)}_{2}L\right]\left[O_{2}^{-}\right]+k_{3}\times [Ni(III{)}_{2}L]\left[O_{2}^{-}\right]$$

(7) $$K_{NiH_{6}L}^{{Ni}_{2}H_{6}L}0$$

(8) $$\frac{d\left[Ni\left(III\right)\right]}{dt}=\;k_{2}\times \left[Ni{\left(II\right)}_{2}L\right]\left[O_{2}^{-}\right]-k_{3}\times [Ni(III{)}_{2}L]\left[O_{2}^{-}\right]-\;k_{4}\times [Ni(III{)}_{2}L]$$

(9) $$\frac{d\left[Ni{}^{*}\right]}{dt}=\;k_{4}\times [Ni(III{)}_{2}L]$$

In agreement with earlier reports, both reactions of the dismutation cycle [Eq. (2),(3)] are assumed to be second order, that is, first order with respect to each reactant.

There are several absorbing species at 260 nm and the absorbance is expressed by Equation 10:

(10) $$Abs=\;\epsilon_{Ni\left(II\right)}\;\times \;\left[Ni{\left(II\right)}_{2}L\;\right]+\;\epsilon_{Ni\left(III\right)}\;\times \;\left[Ni{\left(III\right)}_{3}L\right]+\;\epsilon_{Ni{}^{*}}\;\times \;\left[Ni{}^{*}\right]+\;\epsilon_{O_{2}^{-}}\;\times \left[O_{2}^{-}\right]\;+\;\epsilon_{H_{2}O_{2}}\times \;\left[H_{2}O_{2}\right]\;$$

The concentration profile of each species was obtained by solving numerically the differential equation system [Eqs. (5)–(9)].^[22]^

The experimental data were evaluated by fitting each kinetic trace on the basis of Equation (5)–(9) by using a nonlinear least‐squares routine. The calculations revealed that simultaneous fitting of *k*_2_ and *k*_3_ is not feasible due to strong cross‐correlation between these parameters. When one of these rate constants was included with sufficiently high fixed value in the fitting process, the calculations reproduced the experimental kinetic traces reasonably well. Notably, setting the values of these parameters below a threshold led to unrealistic results for the molar absorptivity of Ni^III^_2_ **L** and *k*_3_ (Table S2). It was established that the best fit (i.e., the smallest standard deviation of the data) was observed when *k*_2_ was fixed at 10^8^ m ^−1^ s^−1^ or higher. In the final fitting procedure, *k*_3_, *k*_4_, *ϵ*(Ni^III^) and *ϵ*(Ni*) were allowed to float, *k*_2_ was fixed at 10^8^ m ^−1^ s^−1^ and *k*_1_ as well as the molar absorptivities of Ni^II^_2_L, O_2_^−^, and H_2_O_2_ were involved with fixed values from independent experiments. The parameters obtained by fitting each trace separately agree within 10 %, confirming the validity of the proposed kinetic model (Table 2). It needs to be emphasized that the experiments were carried out at constant pH that was close to physiological pH. Most of the fitted parameters are expected to be pH dependent and the reported values are applicable only at pH 7.8 (in water/DMSO, 1:1 solvent).

**Table 2:** Kinetic parameters resulting from globally fitting the kinetic data.

Parameter	Value	Unit
*k*_1_^[a]^	(3.86±0.06)×10^4^	m ^−1^ s^−1^
*k*_2_^[b]^	>1.0×10^8^	m ^−1^ s^−1^
*k*_3_	(1.9±0.2)×10^7^	m ^−1^ s^−1^
*k*_4_	215±6	m ^−1^
*ϵ*_Ni(II)_^[a]^	(2.13±0.02)×10^4^	m ^−1^ cm^−1^
*ϵ*_Ni(III)_	(3.19±0.08)×10^4^	m ^−1^ cm^−1^
*ϵ*_Ni*_	(6.60±0.03)×10^4^	m ^−1^ cm^−1^
*ϵ*O_2_^−[a]^	(2.69±0.04)×10^3^	m ^−1^ cm^−1^
*ϵ*H_2_O_2_^[a]^	35±3	m ^−1^ cm^−1^

[a] These values are known from independent experiments and were kept fixed during the fitting procedure. [b] This parameter was held fixed during the fitting procedure.

These results confirm that the first reaction is faster than the second in the dismutation cycle; that is, *k*_2_>*k*_3_. This is a highly unexpected result because earlier studies on related systems suggest that the Ni^III^ form of the catalyst is in the steady state, implying quite the opposite relation between these rate constants. It is an intriguing issue whether the stabilization of the oxidized form is a unique feature of this NiSOD model, or it may occur in other systems including the enzyme itself.

**Figure 6:**
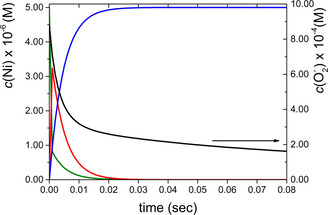
Calculated concentration profiles of each species as a function of time. Green: Ni^II^_2_ **L**; red: Ni^III^_2_ **L**; blue: Ni*; black: O_2_^−^. *c*(Ni^II^_2_ **L**)^0^=5 μm, *c*(O_2_^−^)^0^=877 μm.

The calculated concentration profile for each species as a function of time is shown in Figure 6. The results confirm that the Ni_2_ **L** complex exhibits extremely high SOD activity. About 70 % of the initial amount of O_2_^−^ is dismutated in the first 15 ms of the reaction. Within the same reaction time, the active forms of the catalyst are efficiently removed by the fast degradation process [Eq. (4)] and eventually the Ni_2_ **L** assisted dismutation ceases. The noted slow absorbance decay at longer reaction times is associated with the spontaneous decomposition of the residual superoxide ion.

## Conclusions

In this paper, we report thermodynamic, formation kinetic, structural and SOD activity studies on a new binuclear nickel complex bearing the NiSOD binding motifs. A lysine scaffold was used to connect the HisCysAspLeuProCysGlyValTyr peptide sequence. The metallopeptide exhibits superior nickel(II) binding ability and the formation of the binuclear complex is preferred even in equimolar solution of the metal ion and the ligand. At physiological pH‐range, the major species possesses the (NH_2_,N_amide_,S^−^,S^−^) donor set with square‐planar geometry. This coordination environment corresponds to the active site of NiSOD.

The complex formation kinetics of the mono‐ and binuclear nickel(II) complexes is controlled by the rate‐determining structural rearrangement of the ligand. Binding of the first nickel ion induces further structural changes to accommodate fast coordination of the second nickel(II) ion.

Structural characterization of the Ni^III^ complexes by EPR spectroscopy confirms that the structure of the transient nickel(III) complexes depend on the pH and the metal to ligand ratio. At 2:1 metal to ligand ratio (both NiSOD binding loops are loaded by nickel) an exclusive species was observed and its structure corresponds to the oxidized form of NiSOD in which both metal centers are oxidized to Ni^III^.

The Ni_2_ **L** complex exhibits superior SOD activity, therefore it is an excellent model of the native NiSOD enzyme. However, fast oxidative degradation of the complex eliminates the SOD activity relatively quickly. It is a challenging question why such degradation does not affect the activity of the native enzyme. Unique structural arrangement of the peptide chains, specific features of the medium in biological systems and other factors may be important in this respect. Further studies should focus on the modifications of the model system to improve its redox stability without reducing its excellent SOD activity.

## Conflict of interest

The authors declare no conflict of interest.

## Supporting information

As a service to our authors and readers, this journal provides supporting information supplied by the authors. Such materials are peer reviewed and may be re‐organized for online delivery, but are not copy‐edited or typeset. Technical support issues arising from supporting information (other than missing files) should be addressed to the authors.

Supplementary
