# Supplementary material for: High Enzyme Activity of a Binuclear Nickel Complex Formed with the Binding Loops of the NiSOD Enzyme
Source: Chemistry. 2020 Nov 9;26(70):16767–73. doi: 10.1002/chem.202002706 (PMC7756883; doi:10.1002/chem.202002706)
Supplement: Supplementary file 1 — Supplementary [file CHEM-26-16767-s001.pdf]

Chemistry–A European Journal

Supporting Information

High Enzyme Activity of a Binuclear Nickel Complex Formed with the Binding Loops of the NiSOD Enzyme**

Dóra Kelemen,[a] Nóra V. May,[b] Melinda Andrási,[a] Attila Gáspár,[a] István Fábián,[a, c] and Norbert Lihi*[a, c]

Table of Contents

Experimental Procedures 2
Results and Discussion 3
References 12
Author Contributions 12

Experimental Procedures

Materials. The peptide used in this study was purchased from Synpeptide Co. (Shanghai, China) and used without further purification. The concentration of the peptide stock solutions was determined by pH-potentiometric titrations. According to these titrations, the peptide contains small amounts of trifluoro acetate which is used to cleavage the resin and the protecting groups during the preparation of the peptide. This contaminant has negligible effect on the complexation processes. The $NiCl_2$ stock solution was prepared from the highest available grade (≥ 99.95 %; VWR Int., USA) and its concentration was checked gravimetrically via the precipitation of oxinate. For solution equilibrium and spectroscopic studies, triple deionized and ultrafiltered (Millipore Q system) water was used.

pH Potentiometry. The protonation constants (logK_i) of the ligands and the overall stability constants (logβ_{pqr}) were determined by pH-potentiometric titration method using carbonate ion free KOH solution. The carbonate contamination (less than 0.05 %) was determined using the appropriate Gran functions.[1] In these titrations, 3 mL aliquots of the ligands (ca. 1.2 mM) were titrated either in the absence and the presence of metal ion at 1:1 and 2:1 ratios (I = 0.2 M using KCl, T = 298 K). The headspace over the sample was purged with argon to ensure the absence of oxygen and carbon dioxide. The samples were stirred using a VELP scientific magnetic stirrer and the pH measurements were made using a MOLSPIN pH-meter equipped with a 6.0234.110 combined glass electrode (Metrohm) and a MOL-ACS microburette. Strict experimental parameters were used during the pH-potentiometric titrations in order to ensure that the equilibrium is established after adding an aliquot of the titrating solution to the sample. The system was considered to be in equilibrium when the measured potential remained constant within a 0.03 mV range for 9 s. This experimental protocol takes into account that the complex formation reaction is relatively slow and makes possible the determination of the overall stability constants with satisfactory standard deviations .The pH reading was converted to hydrogen ion concentration as described by Irving et al.[2] Protonation constants and the overall stability constants, $\beta_{pqr} = [Ni_pH_qL_r]/[Ni]^p[H]^q[L]^r$ of the nickel(II) complexes (charges are omitted for the sake of simplicity) were calculated by using the designated computational programs, SUPERQUAD[3] and PSEQUAD [4].

Spectroscopic measurements. UV-visible spectra of the nickel(II) complexes were recorded with an Agilent Technologies Cary 60 UV-VIS xenon pulse lamp spectrophotometer in the 200 – 800 nm wavelength range using the same concentration range as in the pH-potentiometric titrations. The circular dichroism spectra were registered with a Jasco J-810 spectropolarimeter using 1 mm and/or 1 cm cells in the 250 – 800 nm wavelength range. Individual samples were prepared for UV-vis and CD experiments. First, the pH of the sample was adjusted by adding HCl or KOH solution then the headspace was purged with argon for 15 – 20 minutes. Finally, the pH of the samples was checked, and the samples were introduced to the spectrometer.

All CW-EPR spectra were recorded with a BRUKER EleXsys E500 spectrometer (microwave frequency 9.45 GHz, microwave power 13 mW, modulation amplitude 5 G, modulation frequency 100 kHz). An 0.2 mL aliquot of Ni(II) sample solution was introduced into a quartz EPR tube then 0.1 mL DMSO solution containing KO_2 (Acros Organics) was added for *in situ* oxidation. Frozen solution EPR spectra were measured in a Dewar container filled with liquid nitrogen at 77 K. The measured spectra were corrected by the baseline spectrum measured in the same way and simulated using a designated EPR program.[5]

Capillary electrophoresis (CE) and CE-MS experiments. Analyses were conducted using a 7100 model CE instrument (Agilent, Waldbronn, Germany) with UV and MS (maXis II UHR ESI-QTOF MS instrument, Bruker, Bremen, Germany) detection. For CE measurements with UV detection fused silica capillaries of 48 cm x 50 µm id. (Polymicro, Phoenix, AZ, USA) were used. In order to achieve fast separation short-end injection was applied (L_{eff} = 8 cm). UV detection was carried out by on-capillary photometric measurement at 200 nm. Samples were introduced hydrodynamically (50 mbar, 2 s) at the anodic end of the capillary. The background electrolyte (BGE) consisted 50 mM NH_4Ac, pH = 9.0. The applied voltage was +25 kV. The capillaries were preconditioned with BGE for 8 min. OpenLAB CDS Chemstation (Agilent) software was used for both controlling the CE instrument and processing the electropherograms. For the CE-MS measurements, a CE-ESI sprayer interface (G1607B, Agilent) provided on-line hyphenation to the CE instrument. Sheath liquid (SL) was transferred using a 1260 Infinity II isocratic pump (Agilent). MS instrument was controlled by otofControl version 4.1 (build: 3.5, Bruker). For CE-MS determinations the following conditions were applied: fused silica capillary: 100 cm x 50 µm id.; hydrodynamic sample injection (50 mbar, 6 s); BGE: 50 mM NH_4Ac, pH = 9.0; SL: 5 mM NH_3 in isopropanol:water = 1:1; SL flow rate: 7 µL/min; applied voltage: +25 kV. The capillaries were preconditioned with the BGE. MS parameters: negative ionization mode; nebulizer pressure: 0.4 bar; dry gas temperature: 200 °C; dry gas flow rate: 8 L/min; capillary voltage: 3500 V; end plate offset: 500 V; spectra rate: 2 Hz; mass range: 300-1400 m/z. Na-formate calibrant was injected after each separation, which enabled internal m/z calibration. Mass spectra were processed by Compass DataAnalysis version 4.4 (build: 200.55.2969).

Stopped flow measurements. The catalytic activities of the Ni(II) complexes in the decomposition of O_2^- were tested in fast kinetic experiments using an Applied Photophysics SX-20 stopped-flow instrument equipped with a photomultiplier tube as the detector. The kinetic traces were collected using 2 mm optical path length and the experiments were performed at 25 °C. The measurements were carried out in 1:1 DMSO − water mixture, and the instrument was used in sequential mode to circumvent the relatively slow homogenization of the solvent mixture. The first syringe was filled with water, the second one with KO_2 in DMSO while the third one with the complex dissolved in 1:1 DMSO and aqueous HEPES buffer (20 mM, pH 7.8). In the first phase of these experiments, the aging loop was filled with the 1:1 mixture of the first and

second solutions to produce a KO_2 reagent in 1:1 water – DMSO solvent. After 40 s incubation time, this mixture was reacted with the solution in the third syringe and the progress of the reaction was monitored at 260 nm.
The O_2^- solutions were freshly prepared before each experiment by dissolving solid KO_2 (Acros Organics) in vigorously stirred DMSO containing 18-crown-6. The formation of and ion pair between O_2^- and K^+ incorporated in the crown ether improves the solubility of KO_2. This carrier molecule does not affect the kinetic experiments. The concentration of the superoxide stock solution was checked by utilizing the reaction between O_2^- and *para*-nitro blue tetrazolium chloride (NBT) which produces diformazan with characteristic molar absorptivity at 560 nm.

Results and Discussion

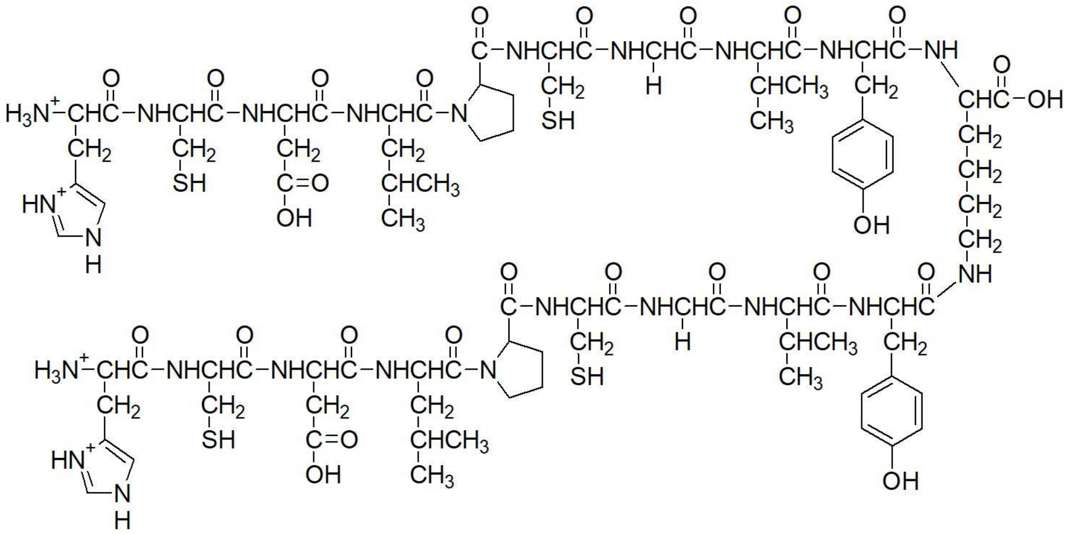

Scheme S1. Structural formula of the protonated binding loops of the NiSOD enzyme (**L**).

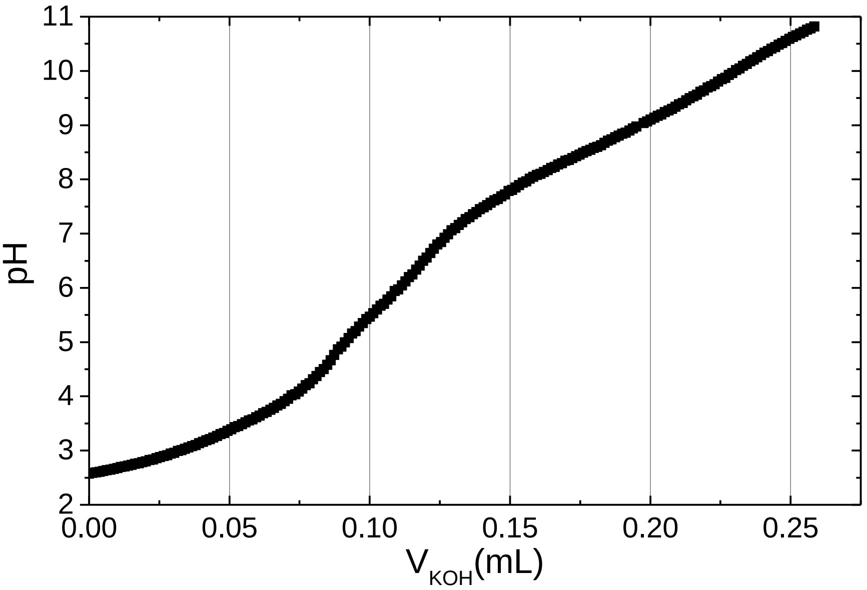

Figure S1. Representative titration curve of the H^+/**L** system. c_L = 1.11 mM

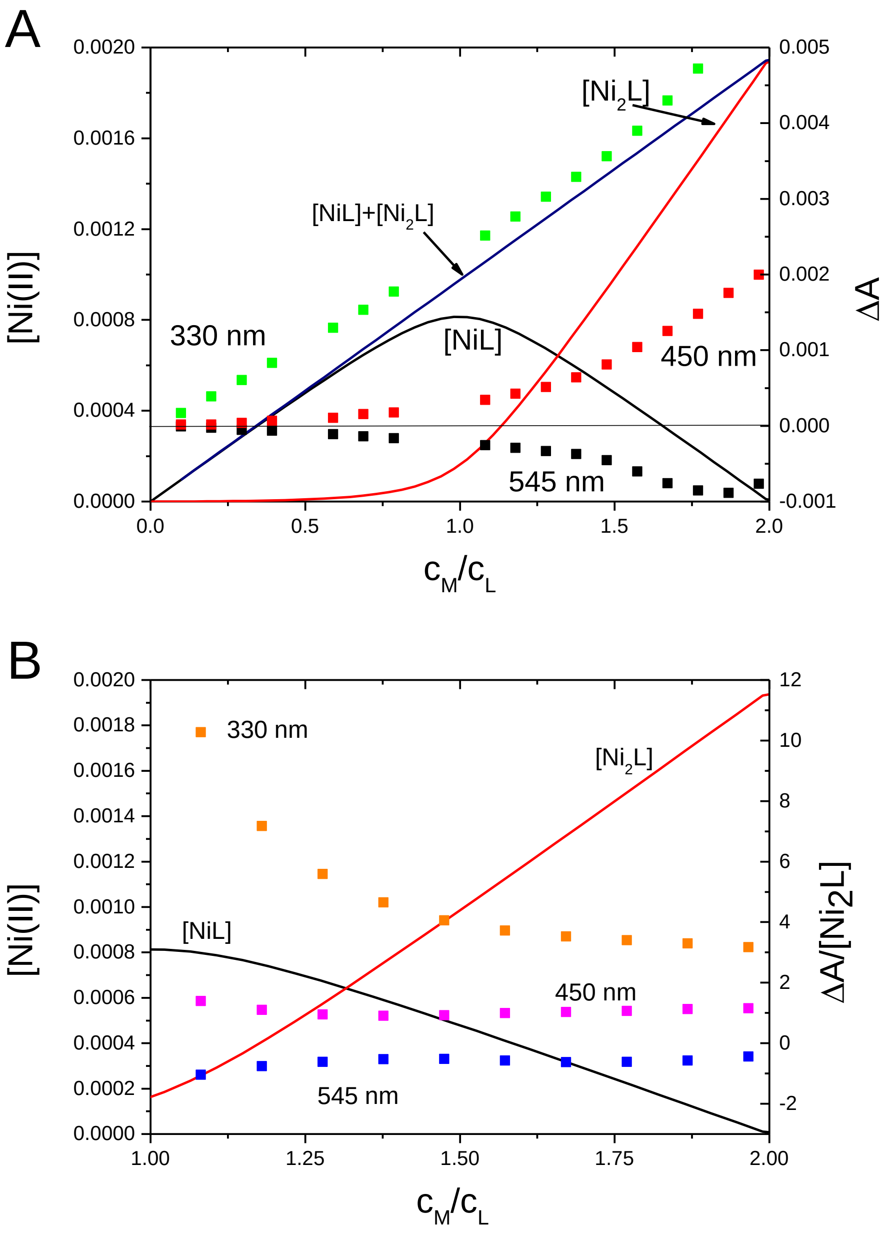

Figure S2. Distribution of the complexes at pH 7.6 and Δ*A* (a) as well as Δ*A*/[Ni2L] (b) as a function of the metal to ligand ratio.

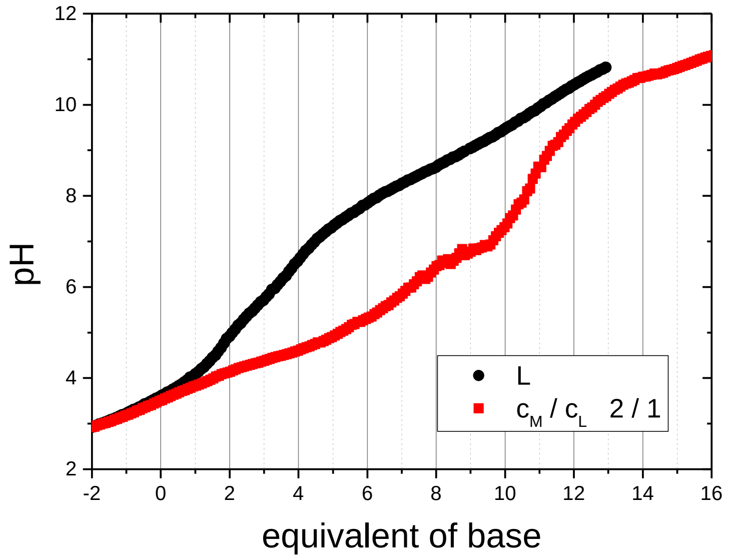

Figure S3. Representative titration curves of the H+/**L** and the Ni(II)/**L** system at 2:1 metal to ligand ratio. Negative base equivalent refers to an excess of acid in the samples.

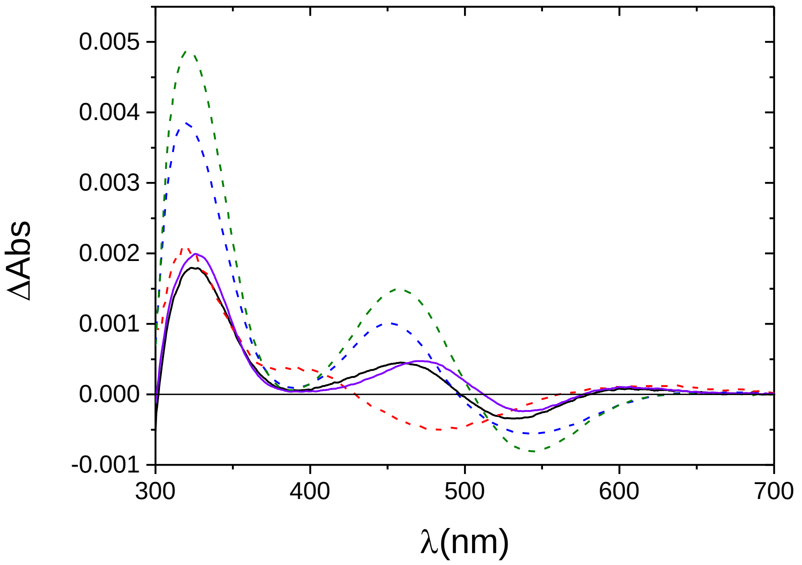

Figure S4. Selected CD spectra recorded in the Ni(II)/**L** system. CD spectra at 2:1 metal to ligand ratio: dashed lines at pH 6.28 (red), 6.82 (blue) and 8.82 (green). CD spectrum at 1:1 metal to ligand ratio, pH 7.62 (violet). CD spectrum of the nickel(II) complex formed between Ni(II) and the wild-type fragment of NiSOD at pH 7.80 (black).

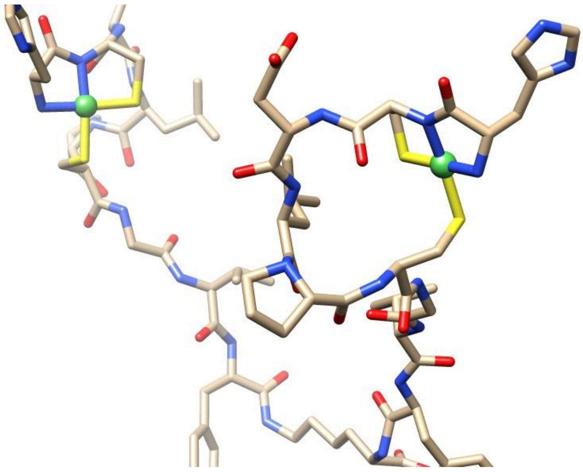

Figure S5. Plausible structure of the reduced form of the Ni_2L complex. H atoms are omitted for clarity.

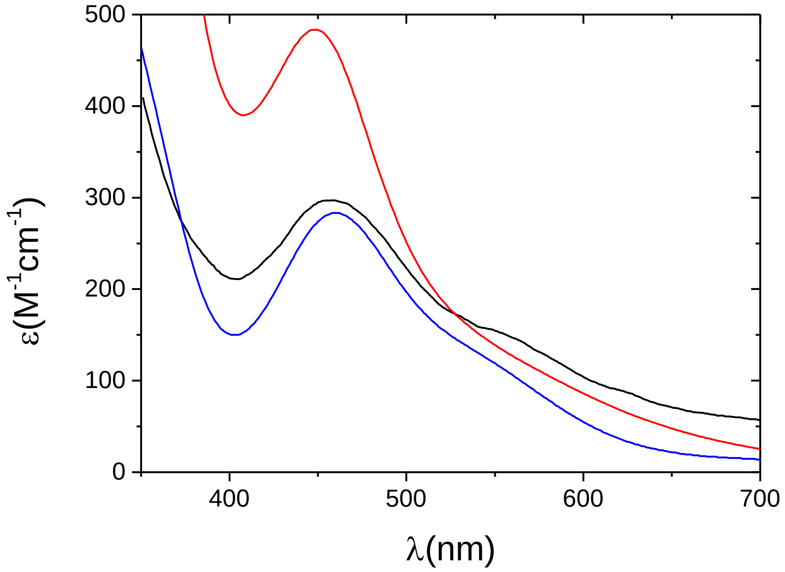

Figure S6. Molar UV-vis absorption spectra of the nickel(II) complexes formed between Ni(II) and **L** at 2:1 (red), 1:1 (black) metal to ligand ratio (pH 7.60) and that of the complex formed between Ni(II) and the wild-type fragment of NiSOD at pH 7.80 (blue).

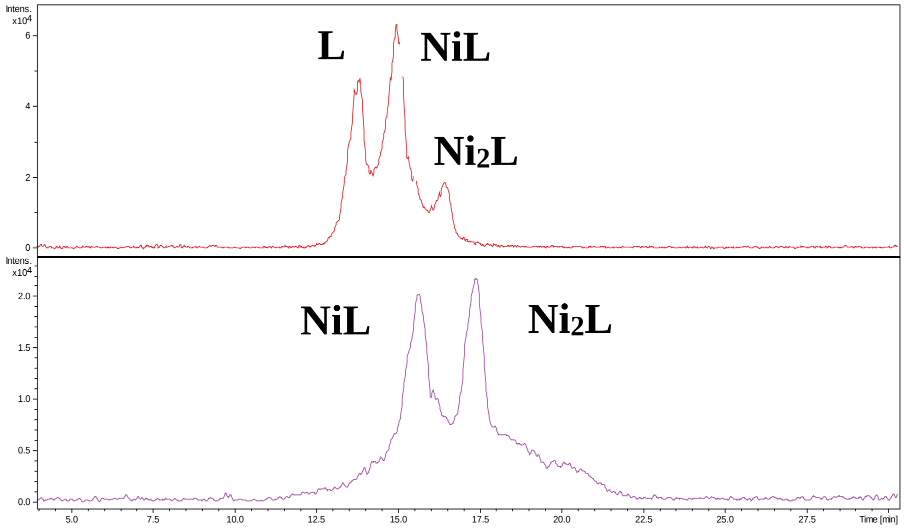

Figure S7. Extracted ion electropherogram obtained with CE-MS at 0.9:1 (top) and 2:1 (bottom) metal to ligand ratio (pH = 9.0). c_L = 110 μM

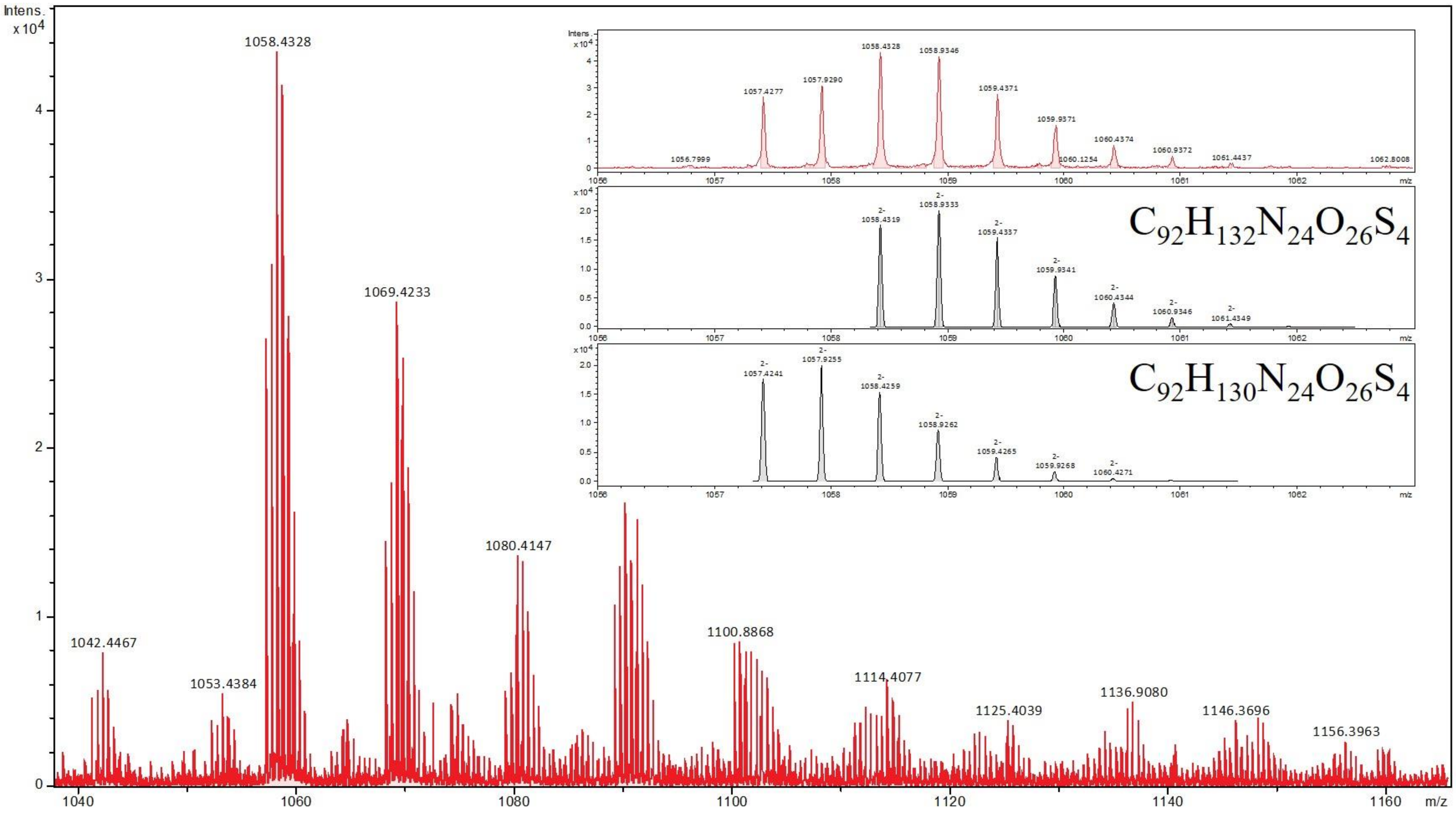

Figure S8. Mass spectrum and calculated isotope patterns (inset) of **L** obtained from CE-MS experiments. The peaks of **L** at 1058.4328 *m/z* are followed by further peaks at 1069.4233 and 1080.4147 corresponding to the stepwise replacement of H^+ by Na^+.

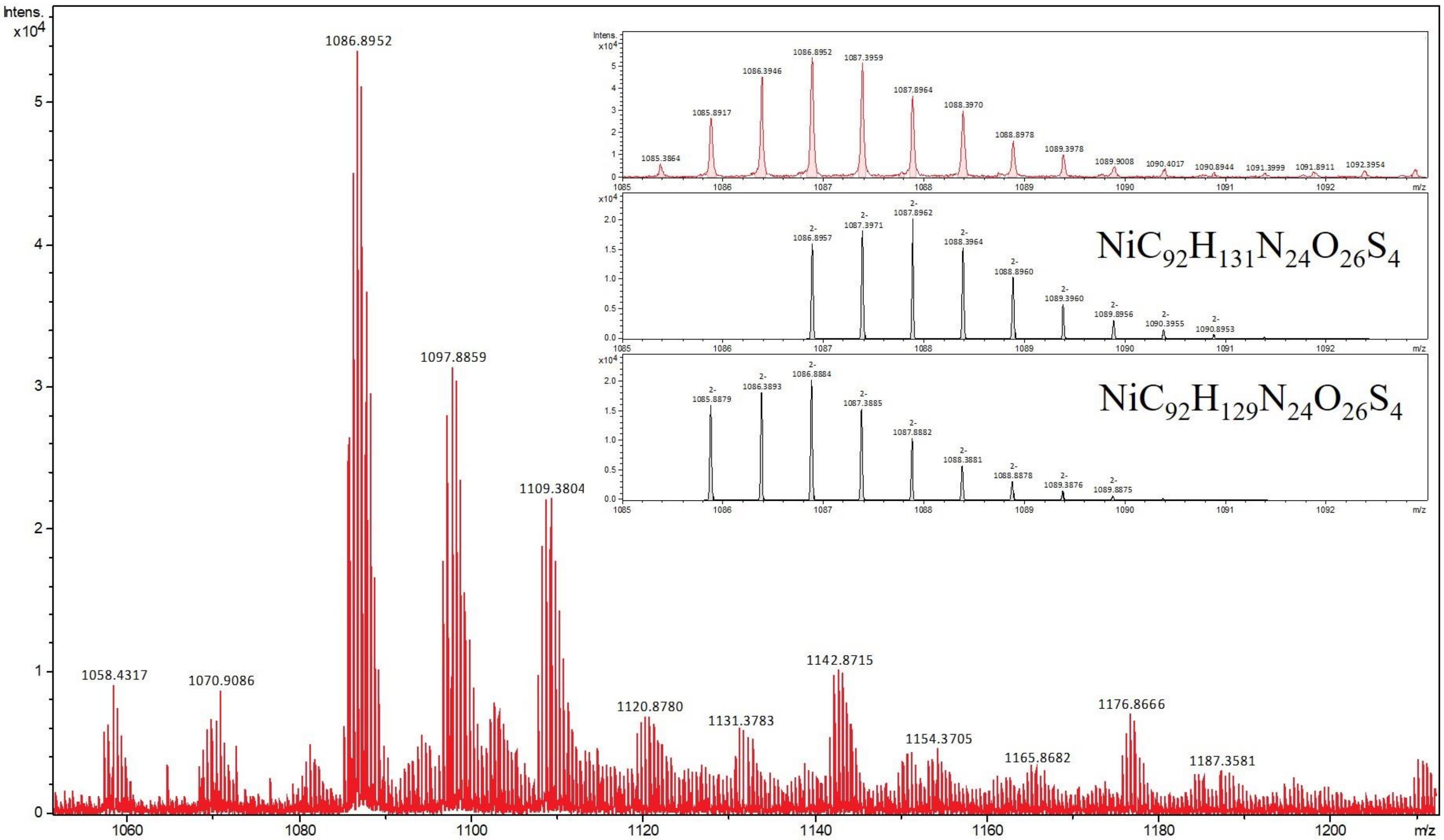

Figure S9. Mass spectrum and calculated isotope patterns (inset) of NiL complex obtained from CE-MS experiments. The peaks of NiL at 1086.8952 are followed by further peaks at 1097.8859 and 1109.3804 corresponding to the stepwise replacement of H^+ by Na^+.

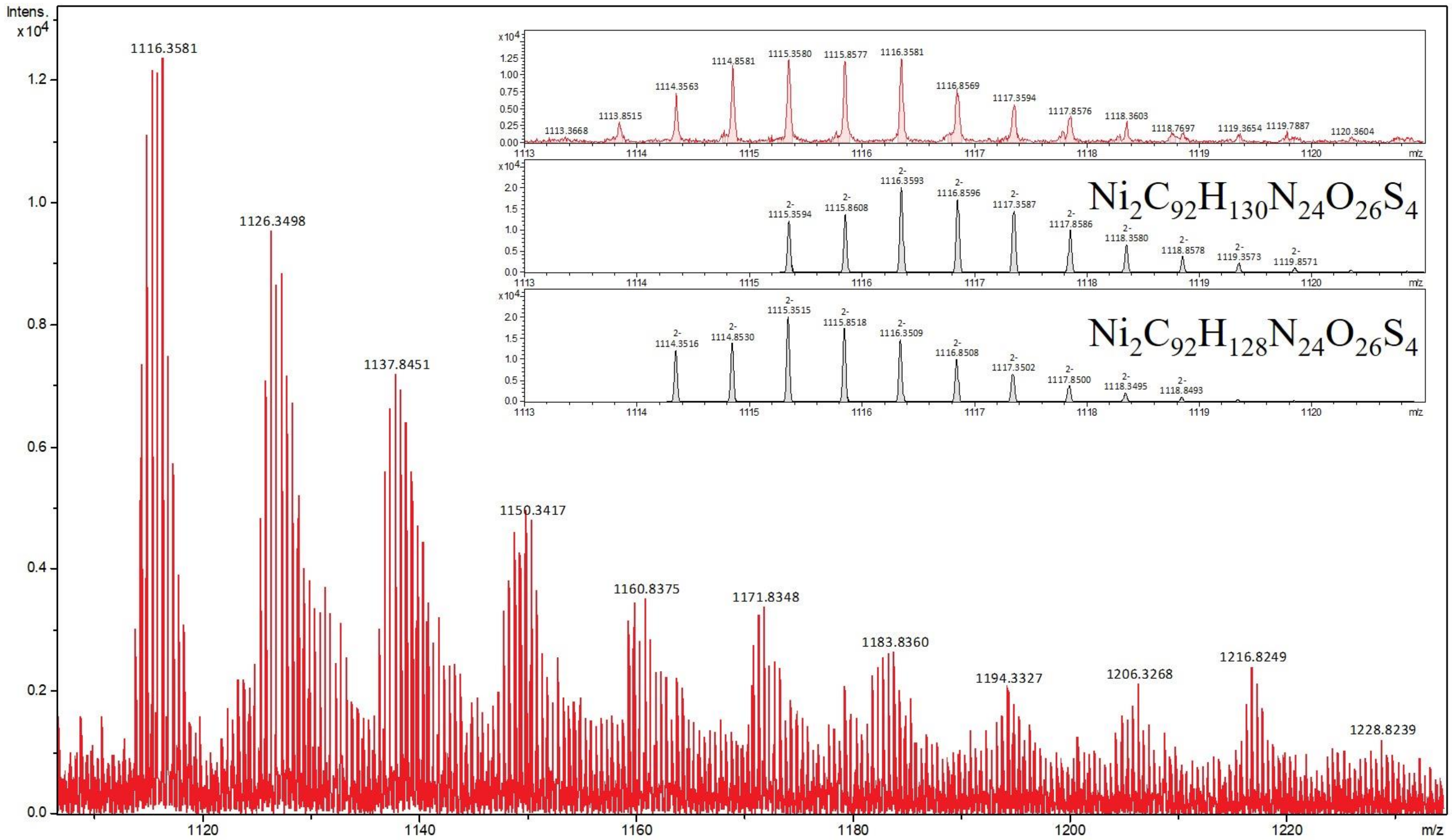

Figure S10. Mass spectrum and calculated isotope patterns (inset) of Ni_2L complex obtained from CE-MS experiments. The peaks of Ni_2L at 1116.3581 is followed by further peaks at 1126.3498 and 1137.8451 corresponding to the stepwise replacement of H^+ by Na^+.

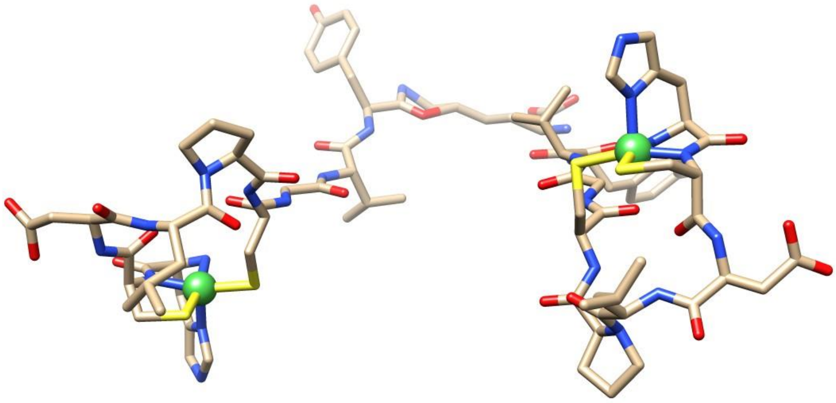

Figure S11. Plausible structure of the oxidized form of the Ni_2L complex. H atoms are omitted for clarity.

Table S1. EPR parameters of the identified nickel(III) complexes.

c_M/c_L	pH	Component	g_x	g_y	g_z	$a^N{}_{x,y}$/G	$a^N{}_z$/G	δ_x/G[b]	δ_y/G [b]	δ_z/G[b]	Comp. ratio/ %
2/1	11.8	comp. 5	2.266	2.199	2.014	20.3	24.8	14.4	17	4.8	100
2/1	7.86	comp. 1	2.287	2.219	2.011	19.9	24.8	14.8	17.1	5	100
1/1	11.8	comp. 3	2.264	2.205	2.015	14.3, 14.3	24, 24	34.2	23.4	3.7	67
		comp. 4	2.259	2.053	1.945			18.1	31.8	15.3	33
1/1	10.54	comp. 1	2.289	2.224	2.012	21.0	23.8	15.5	18.6	4.2	50
		comp. 2 [a]	2.103					100			50
1/1	9.18	comp. 1	2.289	2.224	2.012	21.0	23.8	15.5	18.6	4.2	10
		comp. 2[a]	2.103					100			90
1/1	9.02	comp. 1	2.286	2.220	2.012	21.3	23.8	15.1	21.2	3.6	100
1/1	8.01	comp. 1	2.284	2.226	2.012	18.9	24.7	18.3	16.5	4.7	100
wtNiSOD [c]			2.289	2.220	2.012	17.5	25.2	11.8	14.3	4.3	

[a] Component 2 was treated with isotropic *g*-tensor, [b] δ_x, δ_y and δ_z were used to describe the orientation dependent linewidth. [c] Data are taken from Ref. 6.

Table S2. Kinetic parameters and molar absorptivites obtained by fitting one of the kinetic traces shown in Figure 5. Rate constants k_2, or k_3 were involved with fixed values in these calculations. [a]

Parameter	**I.**	**II.**	**III.**	**IV.**	**V.**	**VI.**
k_1 ($M^{-1}s^{-1}$)	3.86×10^4 [b]	3.86×10^4 [b]	3.86×10^4 [b]	3.86×10^4 [b]	3.86×10^4 [b]	3.86×10^4 [b]
k_2 ($M^{-1}s^{-1}$)	1.00×10^8 [b]	1.00×10^7 [b]	1.00×10^6 [b]	$(1.8 \pm 0.4) \times 10^7$	$(1.9 \pm 0.5) \times 10^7$	8.7×10^8
k_3 ($M^{-1}s^{-1}$)	$(2.05 \pm 0.05) \times 10^7$	$(2.5 \pm 0.5) \times 10^7$	$- 4.8 \times 10^6$	1.50×10^8 [b]	1.00×10^8 [b]	1.00×10^7 [b]
k_4 ($M^{-1}s^{-1}$)	206 ± 8	205 ± 59	4.4 ± 0.2	1530 ± 20	1010 ± 10	575
ε(Ni(III)) ($M^{-1}cm^{-1}$)	$(3.11 \pm 0.03) \times 10^4$	$(3.10 \pm 0.02) \times 10^4$	$- 4.2 \times 10^4$	$(6 \pm 2) \times 10^4$	$(5.0 \pm 0.1) \times 10^4$	$- 2.8 \times 10^5$
ε(Ni*) ($M^{-1}cm^{-1}$)	$(6.61 \pm 0.02) \times 10^4$	$(6.62 \pm 0.04) \times 10^4$	5.5×10^4	$(6.62 \pm 0.02) \times 10^4$	$(6.62 \pm 0.02) \times 10^4$	3.9×10^4
SD	0.016	0.057	0.143	0.161	0.076	0.226

[a] Solvent: 1:1 aqueous buffer of HEPES (20 mM, pH 7.8) / DMSO mixture. λ = 260 nm, 2 mm optical path, c_{Ni_2L} = 5 μM, $c_{O_2^-}$ = 877 μM [b] The parameter was kept fixed during the fitting process.

References

[1] G. Gran, *Analyst* **1952**, *77*, 661-671.
[2] H. M. Irving, M. G. Miles, L. D. Pettit, *Anal. Chim. Acta* **1967**, *38*, 475-488.
[3] P. Gans, A. Sabatini, A. Vacca, *J. Chem. Soc., Dalton Trans.* **1985**, 1195-1200.
[4] L. Zékány. . I. Nagypál, "Computational Methods for the Determination of Formation Constants," in *Computational Methods for the Determination of Stability Constants*, D. Leggett, Ed., pp. 291–299, Plenum Press, New York, NY, USA.**1985**.
[5] A. Rockenbauer, L. Korecz, *Appl. Magn. Reson.* **1996**, *10*, 29-43.
[6] N. Lihi, G. Csire, B. Szakács, N. V. May, K. Várnagy, I. Sóvágó, I. Fábián, *Inorg. Chem.* **2019**, 58, 1414-1424.

Author Contributions

D. K. was responsible for the sample preparation. EPR experiments and the analysis of the spectra were carried out by N. V. M. CE experiments were performed by M. A. and A. G was carried out the CE-MS experiments. A. G. contributed to the discussion of CE and CE-MS analysis. I. F. and N. L. evaluated the results and wrote the paper. All authors added their respective expertise to the paper.
